# Abstracts and Gleanings

**Published:** 1878-09-20

**Authors:** 


### *ABSTRACTS AND GLEANINGS.*

### MALARIAL AND YELLOW FEVER.

**(An Extract, from the Paper of Dr. Le Hardy, of Savannah, in Transactions Georgia Medical Society, the following.)**

It is pretty generally admitted, by medical scientists, both in America and Europe, that malarial or paludal fevers are produced by plants, or spores of plants, growing in marshes, stagnant water, or elsewhere, whetner at the level of the sea, or ten thousand feet above it, in high, or in low latiudes. With my present imperfect knowledge of the various plants producing febrile diseases, it would be impossible to say, whether those producing malarial fevers belong invariably to the fungi order.

Lebert says: "It is true, that in diptheritis, vaccinia, small-pox, septicæmia, pyæmia, mycosis intestinalis and in lymph thrombi of puerperal diseases, the globular form of protomycetes prevails. The fact, that Davaine has already demonstrated, the constant presence of rod bacteria in malignant pustule; and that the spiral-like filaments of Obermeyer are found in relapsing fever, prove that the spiral protomycetes may exist in infection, as well as the globe, rod-like, or ovoid, or as the panhistophiton of the silk worm, discovered by me."

But the "protomycetes" of Lebert propagate by "gernmation," and not by fructification. The result of this is, that the diseases produced by them require either contact, as in vaccinia, pustule syphilis, or absorption into the system by the stomach, through infected water, food, etc., as in typhoid fever, or to change their form when leaving the body in the ejecta, therein completing their growth, and producing new germs or plants, which being disseminated through the surrounding air, enter the body. This is believed by many, to be the mode of propagation in cholera. At any rate, Kock has demonstrated that such changes take place in the bacillius anthracis of splenic fever.

The history of yellow fever, as well as of malarial fevers, points to a different mode of origin and propagation. They are peculiar to certain localities; have a well defined duration; they always recur under the same conditions, and always disappear under the influence of frost.

The whole atmosphere must contain the germs of these fevers, in order to account for the almost simultaneous appearance of fellow fever, in widely separated portions of a city, and of malarial fevers, over a large expanse of country. Spores of fungi, floating in the air, have frequently been collected, and made to produce the plants of their kind. I have often, after collecting the spores of the mould plant, so common in this locality in summer, watched its growth under the microscope.

These spores are exceedingly light, and are carried up from the earth by the effects of solar heat, when they are wafted about by the winds.

In the same manner, the spores producing yellow fever, rise from the surface of stagnant water or wet soil, and are carried along by the winds until they find a lodgment, in a city, a house, a ship, or other place. Here, by constant additions from the same source, they may concentrate sufficiently to produce effects, more or less poisonous, in proportion to the quantity absorbed.

It is a well known fact, that malarial fevers have been contracted by persons passing through marshes, or sleeping in the neighborhood of these, or other malarial spots. It has also been generally observed that men, who are more exposed to the poisoned air of a city, are much more frequently stricken, during an epidemic of yellow fever, than women, who usually remain at home.

The question next arises, whether the different forms of malarial fevers are produced by spore plants of the same, or of different species. This I deem necessary to discuss now; but it may be well to remark that intermittent fevers exist in the localities where remittent fevers prevail, and that both of these are particularly rife in sections visited by continued fevers and yellow fever; also, that the variations which take place in the nature of these fevers, occur *pari passu*, with the increase of temperature.

It is a fact beyond dispute, that where the air of houses, ships, etc., has been allowed to remain undisturbed by disinfection, or by frost, sporadic cases of yellow fever have originated therein; and although intermittent, or remittent fevers were prevailing in the surrounding localities, the yellow fever did not spread beyond the source of infection. This inclines me to the opinion that the disease under consideration is the product of a plant, which can fructify only at a temperature of about 80°, and I think I am sustained in this view of the subject, by the following experiments made during the months of February and March, of this year (1878).

Having collected with great care, during our epidemic, three specimens of black vomit, one of urine, one of blood, one of liver, and two of the dust floating in infected houses, and having kept them sealed, I instituted experiments. These I made under glass bells that had been subjected to a heat of over 250°, to which heat the stand and jar underneath had also been subjected. The water used was first distilled, then boiled; and in order to prevent the access of surrounding air, or contamination from foreign germs, was introduced into the jars, through a previously heated and purified syphon, by which means the specimens were passed into the jars twenty-four hours afterwards.

The results obtainen so far are of such a nature as to make me hopeful of bringing to light, at some future day, the *punctum desideratum* in the history of yellow fever.

In two specimens of black vomit, and in one derived from infected dust, I have obtained several plants of a fungus which I believe is peculiar to yellow fever, and which has not, so far as the light of my researches enables me to judge, been described before. The other specimens gave negative results. On the fluid containing the three specimens just mentioned, dark specks filaments shot out, and spread rapidly on the surface, and in two days the plant had attained its full growth, extending in a circular form, some fourteen lines in diameter. To the naked eye they appeared like very finely woven spider webs. On the third day, extremely fine projections or stems of about a line in height, could be perceived with a lens. On the next day rounded swellings (sporangia ?) were discernable at the extremity of most of the stems. Here, however, the progress of growth ceased, and during the fifth day the spore bearing ttems drooped down, and on the sixth day, the plant sank underneath the surface of the water without having matured spores.

Under a 1-5 objective, the mycelium was seen to be composed of innumerable filaments, growing primarily from a common centre, branching off at intervals, and interlacing with each other.

They were formed of elongated cells, destitute of chlorophyll. The stems also were composed of tapering cells. The spore cases were of an oval form, pointed at the upper extremity, and apparently consisting of several layers of cells, in which was contained a transparent fluid. In no instance did I discover *one*, containing granules or spores.

The mean temperature of my office during the time at which these experiments were made, was 69° in the shade, and I am led to believe from these incomplete experiments, that the yellow fever fungus cannot complete its growth and fructify at a temperature much less than 80° F.

For reasons as follows :

I. Because, it belongs exclusively to malarial countries.

II. Because, I believe its cause to be spores of fungi floating in the air.

III. Because, in violent cases terminating in black vomit, there are instances of indubitable remission or intermission in the fever, and in lighter cases these symptoms are frequent.

IV. Because, in my treatment of the negro population ninety per centum presented the remittent or sometimes the intermittent form.

V. Because, relapses occur as in all malarial fevers.

VI. Because, in the majority of cases, the fever is controllable by the use of sulphate of quinine.

---

### THE OPIUM HABIT.

Dr. Gould, in "Medical and Surgical Reporter" says :

In considering the matter of cure of the opium habit, let the following points be kept constantly in remembrance :

1. No other drug or combination produces like effects upon the system, therefore nothing else can entirely supply its place.

2. Each preparation of opium possesses its own peculiar power, which differs in a marked degree from the power of every other preparation. For example, if an individual is addicted to the use of morphia, no other preparation of opium, though containing an equivalent quantity of morphia, will supply its place. It will already occur to the reader that opium *must be used* in the cure of the habit; to contend otherwise is evidence of ignorance of the practical phase of the subject. Now, I gradually withdraw the narcotic, but the quantity withdrawn must be compensated for in some way. After years of effort and trial I have discovered that no article will so successfully act this part as nux vomica, and that this may be so combined as to be much more effective than the drug alone. I have not the time to notice all the conditions of the opium eater, and the indications to be met, but shall at once indicate my mode of treatment, which, if associated with sufficient will power on the part of the patient, will be successful in every case.

I prepare an elixir as follows: Dissolve 96 fl ounces aqua dest. 12 troy ounces of sodium phos. and 512 grs. val. ammo., then add to the solution 112 fl dr. dilute phos, acid, and 2 fl oz. hydrochloric acid. Now, exhaust, by percolation, with stronger alcohol (8 fl oz.), 1024 grs. *pure* powdered nux vomica, and 160 grs. aromatic powder, U. S. P., driving through the last portions of the menstruum with aq. dest. until it commences to pass cloudy. Mix the pecolate with the acid solution of sod. phos.; add to the mixture 16 fl oz. glycerin, filter through paper, and if the measure is short of one gallon, wash the filter with sufficient distilled water to supply the deficiency.

Though I have been using a similar combination for years, for the abo ve for mula I am indebted to Dr. Green, of the firm of Chapman, Green & Co., manufacturing chemists, Grand Crossing Illinois.

Now, how do I use it ? Suppose that morphia is the preparation taken. In one pint of the elixir I dissolve the amount of the narcotic taken in one month, less 25 per cent., and direct my patient to take dr. j. four times a day. If the quantity taken is immense, say 20 or 30 grs. per diem, or the system is badly shattered, with much digestive trouble, or intestinal apathy, I double the dose of the elixir.

After the first month I reduce the opiate 20 per cent., monthly, and the prtient is not aware of the reduction until the quantity of morphia is comparatively small, depending, of course, on the quantity to which the system had been habituated. But, should there be rebellion, it will likely be slight, requiring only will power to subject it. In some cases it may be necessary, toward the close of the treatment, to reduce less rapidly, yet I have never reduced less than 20 yer cent. The point at which the narcotic is left out, the pure elixir only being given, will depend on circumstances, such as quantity taken, etc. In case of sleeplessness, a full dose of bromide of potassium or sodium will usually produce quiet and rest. In some cases, perhaps, chloral will be required, but, if so, it must be given only when absolutely necessary. I have never been compelled to resort to it.

Another matter I will refer to briefly. It is necessary that the patient be as actively employed as the strength will admit; as idleness and constant thought of the habit will retard the cure. Every physician will understand this. And another thing is necessary. In the beginning of the treatment the victim must be determined to succeed, as each successive effort at redemption will meet with a weakened will power.

I have not the time to specify all the details of treatment, but they will naturally be suggested to the intelligent physician. It must be remembered, however, that opium is a powerful agent in producing hallucinations, and much of the difficulty (if not actual pain) experienced can be dissipated by the exercise of a strong will.

In conclusion, I will say to the many physicians who have deluged me with letters, that sickness has prevented me from concluding these articles sooner, and also accounts for the rapid disposal of the subject. If, however, the directions I have given are faithfully followed, the most confirmed opium eater can be redeemed, and be redeemed with ease.

---

CHRONIC INTERMITTENTS.

In the therapeutics of intermittent fever it is not enough when a patient presents himself, to diagnose the disease and prescribe for the name on a routine plan. The history, temperament, habits and collateral derangements must be carefully scrutinized, and the remedies as well as the doses precisely adapted to them. It is also necessary to erase as much as possible from the mind the dominant notion of a malaria in the system, and instead, to fix the attention *searchingly* upon all the organs whose functions are most seriously implicated. To correct these, after the paroxysms are for the time arrested, is the *sine qua non* of ultimate success. This, as a rule cannot be accomplished by continuedly active or disturbing medication, but only by that which will hold the organs most at fault up to the standard of healthy action, and no more; neither causing them to sink below their normal activity nor to rise much above it. Accomplish this successfully—guarding the tendency to periodicity by quinine at proper intervals, and the health will soon be toned above the ague point and the tendency to relapse permanently obliterated.

In carrying out these principles of treatment, the exact course adapted for all cannot, of course, be laid down, as this is a matter of individuality, scarcely any two persons exhibiting the same phases and modifications of derangement.

But suppose a person suffering by a chronic intermittent, shows something like the following symptoms: The disease has not been on him longer than at frequent intervals for four or six months; the nourishment and strength of the body are fair, conssdering the antecedents, but there is a sallow or dusky hue of the skin; the eye is heavy and listless, the tongue heavily coated, the lips moderately anæmic, the appetite capricious, the bowels irregular, with vertigo or cephalalgia more or less constantly, the renal secretion is now highly concentrated and tinted with bile, then clear and abundant, with aching in almost every hone of the body which is now better, now worse; and withal, severe paroxysms of chills and fever on alternate days. The prompt arrest cf these paroxysms is indispensible, as it is these which cause, aggravate and perpetuate the organic derangement, especially of the chylopoietic viscera, until their functions are always, even during intermission, very imperfectly performed; thus depraving the assimilative functions and the organization of the blood. It is this and nothing more, which is at the root of the so-called malarious cachexia, and in proportion as bad management, not imbibation of malaria, is prolonged, does the certainty as to the appearance and degree of the cachexia, *ceteris paribus*, mainly depends. I therefore, make it a rule never to allow a person to have a paroxysm after being seen, if I can help it, although in most instances the action of quinine is not only less certain but far more unpleasant in its effects if given without preparatory treatment. In most instances this only consists in the administration of an efficient cathartic of podophyllin and mercury. When there is a diarrhœal tendency leptandrin acts with the mercurial to better advantage than the podophyllin. After the viscera are relieved of their loaded, vicious secretions, the quinine should be administered, and as a rule one-third less is required to stop the paroxysms than if that had not been done. Ten or twelve grains are sufficient in one case, and from fifteen to twenty in the other. After the regular paroxysm is arrested no more quinine need be given until the seventh, fourteenth and twenty-first days, unless a gross indiscretion of the patient should cause it to return sooner. On these days, the same amount is to be taken as in the first instance—say three grains before each meal, and at bed time until the requisite amount is taken.

The treatment of the intermission is highly important, it is in fact the grand requisite of success. If the tongue, after the catharsis, remains coated—with sluggish peristalsis and icterode hue of the conjunctiva or skin, I order the following:

R.—Protoid. mer ................ grs. ii.
Podophyllin ................ grs. i.
Nit. pot.... ................ grs. vi. M.
Make four powders.

to be given one at bed-time of each day until the appearance of the tongue and skin are normal. The reason for administering the medicine at that time is this: the night hours allow the remedies to act therapeutically in an undisturbed manner by either food or drink. As soon as the tongue is clean and the tendency to hyperœmia and hypertrophy of the liver, and the tumid abdomen is made to disappear, the powders are to be stopped, and resumed if the symptoms reappear.

At the same time or at least as soon as the most extreme chylopoetic derangement is corrected in order to hold these organs up to the standard of healthy action—not to correct any apparent derangement—the following should be administered:

R.—Fl. ex. euonymus ........... oz. ij.
Fl. ex. tarax.
Fl. ex. xanthoxyli.......... aa oz. i. M.

And order a teaspoonful before each meal except in the days when the quinine is to be taken.

When the tendency to revert to marked hepatic torpor is very great, ten or fifteen drops of the fluid ext. stillingia should be added to each dose—or if the spleen is enlarged a few grains of iodide of potash to each dose of the solution will do excellent service. If the patient is in anywise anæmic, the liquid oxy-nitrate of iron should be administered. It forms an excellent solvent and vehicle for the quinine, tones the appetite, facilitates the hematosis, and acts favorably upon the funcions of the liver.

The precautions to be observed by the patient are important. The diet must be simple and plain—no pastry, warm bread, rich puddings, or any highly seasoned complex dishes. No wine, beer or liquor to be allowed. The clothing should be adequate, especially for night protection, or after sudden meteorlogical changes. For these purposes flannel underclothing should be enjoined in all cases in all seasons. The exercise should be mild—never being carried to the point of exhaustion until convalescence ends.

In a few words, the vital strength needed for recuperation should not be spent in exhausting toil. If it is, ten to one the most skillfully directed treatment will prove of only temporary advantage. Ordinarily, the treatment and these observances should be strictly practiced for at least a month, after which a gradual cessatien and a return to ordinary habits may be allowed. It is certainly far better economy to do this for a month than to be partially disabled for several months or even for two or three years, as all experienced observers have frequently seen.

To prevent an intermittent becoming chronic—a teaspoonful of the extracts above named should be taken before breakfast and dinner, after the paroxysms have been arrested with quinine; anticipating the tendency to recurrence on the 7th and 14th days by the latter means. The diet and habits should also be made to conform to the rules already given. In this way there is no difficulty in avoiding a return of the paroxysms every week, or for months.

In inditing these directions there has been no thought of simply filling or rounding out this article according to a routine formula, but every sentence is meant to convey indispensible requirements, not to be simply accepted as sound or sensible directions, but such that must be put in the strictest practical operation for entire success.

The period of treatment, of course, depends upon individual peculiarities, upon the facility with which the reversion to health occurs, upon the extent and profundity of the organic derangement, and upon the length of time which the disease has existed either through mismanagement or the obstinacy of the patient, and upon the fact whether the person is acclimated or not. One of the lymphatic temperament needs treatment longer than one of the sanguine—while those of the bilious lymphatic longer than either.

Such is the outline of treatment for chronic intermittents that I have pursued for years, and with perfectly satisfactory results. I have had patients with enormously enlarged spleen, enlarged liver, tumid abdomen, dirty-pale complexion—who had been using quinine and prusiate of iron for months, interspersed with cathartic pills every now and then, and only with the most transitory benefit, in fact upon the whole, getting worse and worse, or falling into what is termed the malarious cachexia—and had them improve under the plan above given almost uninterruptedly until perfect health ensued.—*Dr. Black in Lancet & Clinic..*

---

REMOVAL OF CUBOID BONE FOR TALIPES-EQUINO-VARUS.

Mr. Richard Davy has now practiced excision of the cuboid bone for relief of talipes-equino-varus, in all, nine times, and with almost uniformly satisfactory results. Before operating on his last, which I saw, he remarked to the class, at the Westminster Hospital, that while his success in the operation had been all, perhaps, he should desire, he would still reserve the procedure for the severer cases of the affection. He did his first operation on the living subject in 1874. His mind was, perhaps, as much directed to the procedure as a possible means for the cure of this variety of talipes by a case which occurred in the practice of his colleague, Mr. Barnard Holt, where, in a twelvemonth after the cuboid bone had been removed for caries unattended with club-foot, a talipes valgus, the antagonistic variety to varus, occurred. The loss of the bone in nowise interferes with locomotion. The injury inflicted on the outer wall of the foot heals kindly, while the subsequent treatment of the case by instruments is simplified and facilitated in every way.

Mr. Davy first saw this patient in 1874. He was then fourteen years old, and had been almost uninterruptedly in the hands of orthopedic surgeons. After having a plaster-cast taken of the foot, Mr. D. excised the cuboid bone in January, 1875. The benefit which ensued was very great, and the contrast between the shape of the foot, as shown by the cast before and after the operation, was most striking; yet, the member was not exactly all that could be desired, and Mr. D. thought it might be much improved by the removal of a wedge from the tarsal remnant, so as to make the patient absolutely a plantigrade, and to banish, in future, all surgical treatment and instrumentation. Mr. Davy narrated, in a few words, his experiments on the removal of the cuboid bone in 1873—how he had practically demonstrated that an accurate excision of a tarsal wedge was required for a complete cure, and while admitting that prolonged instrumentation, cutting tendons, manipulation, etc., would correct distortion temporarily, yet, so surely as a urethral stricture recurs, so also would the deformity until the bone assumed permanent misshape. Hence, he thought it the duty of the surgeon to strike at the root of the matter by rectifying bony distortion, and by osseous union preventing its recurrence. The lecturer added that he much preferred one large operation to a host of smaller ones, and he was able to say now, after his ninth case, that the results of attacking the tarsal arch (osseous), for the cure of club-foot, were sufficiently gratifying to insure their repetition.

The patient being chloroformed, and Esmarch's bandage applied, the foot to be operated on was put in a stationary iron vice, the inside of the jaws of which were covered with cork, so as to insure against injury of the structure. He then cut directly down on the outer aspect of the foot to the cuboid, dividing the indurated skin and bursa. A second incision, extending toward the dorsum of the foot, gave a T-shaped cut. Two stout wires were now inserted, one into each flap, and used as retractors. The upper and outer surfaces of the cuboid were then completely exposed, and, with a chisel with a very thin blade, the necessary wedge was carefully cut from the bone. The wound was closed by several sutures. No dressing was applied further than an internal foot-and-leg splint, secured by a gum and chalk bandage. The operation completed, the foot was brought at once, and without difficulty, into its proper line, and, without some untoward result, the lad will soon walk out of the hospital a plantigrade.—*Dr. Yandell in Practitioner.*

---

### HEMORRHAGES.

We make the following extracts from a paper read by Dr. Bartlett before the Buffalo Medical Association:

#### EPISTAXIS.

The means I have found singly and combined to be an effectual remedy for epistaxis is pressure external or internal. The experience of more than twenty years has convinced me that the ordinary forms of styptic treatment—or packing as ordinarily practiced are wholly unreliable in cases at all severe, or appearing in the course of disease.

In June 1863, I had charge of a case of typhoid fever occurring in a phthisical young gentleman residing on Seneca street, near Michigan. The disease was protracted and the patient became exceedingly feeble from diarrhea and loss of digestive power. Early one morning, I was called to find him bleeding from the nose to a most alarming extent. I used styptics, plugged the anterior nares, and with the assistance of an eminent physician, now deceased, tightly closed the posterior nares—all those proceedings failed to do more than check,—they did not stop the hemorrhage. In certain positions the loss, drop by drop, passed outward, in others inward. General treatment had no perceptible effect, and after 36 hours perseverance I found my patient rapidly failing. In this dilemma I removed all the packing from the nares, when the slow drop, drop, passed outward. At last by pressure applied to the angular branch of the external carotid, I had the satisfaction of finding the hemorrhage fully controlled. This I accomplished by application of the thumb and finger at the lower border of the Ossa Nasi externally. Temporary removal of pressure was followed by hemorrhage, and to secure continuous pressure, I shaped the jaws ot a spring clothes pin to fit the nose, removing a portion of the wire spring to lessen pressure. This simple tourniquet remained in place and the patient recovered. Satisfied, by this experience, of the doubtful utility of packing in the usual way, I felt quite safe until a case of nasal hemorrhage occurred in a patient about 14 years old, residing on Scott street, near Washington. The lad had suffered previously, and his mother did not feel alarmed until he fainted from loss of blood. Before my arrival he slightly rallied only to sink exhausted by fresh bleeding. He was pulseless, sightless, breathless, and to all appearance dead. I forced some whiskey down his throat and practiced artificial respiration. As he gasped, I passed a sponge tent, intended for other use, into the right nostril from which I supposed the blood had flowed. It soon expanded and filled the nostril posteriorly and anteriorly, completely controlling the hemorrhage. I left the tent in position forty-eight hours; on removal, there was no loss of blood, and I had the pleasure of being assured years afterwards that the treatment had been radically successful. Since that date, August 1863, I have used the sponge tent in all alarming cases of hemorrhage of the nose. My cases number 27, comprising those occurring in Typhoid, Diptheria, Scarlatina, troumatic injuries and causes not capable of tabulation. I think I may fairly claim originality in this proceeding.

Post partum hemorrhage, though occasionally accidental, is usually avoidable. Position, and more particularly, deference to nature's mode of placental expulsion would largely diminish the dangers encountered. The reprehensible practice of delivering the Placenta by traction upon the cord cannot be too severely condemned. I have for years practiced what is now known as Crede's method, and always bring the placenta into the vagina by external manipulation; traction upon the cord is then useful and justifiable. The rude separation of the placenta from the uncontracted uterine wall is one of the most frequent causes of undue and dangerous flooding.

The use of sponge tents for epistaxis, dating back no less than eighteen years is original with myself so far as I can learn. Two English physicians in '74, I think, allude to their use; but no work, unless it be very recent, makes any allusion to them for this purpose. I can recommend them as truly specific in such cases. Their size should vary as also their length. They should always pass well to the posterior nares, and be left from forty-eight to seventy-two hours in position; contrary to supposition the withdrawal is quite painless. The error, if any, will be in using tents of too great diameter. I have never used one of more than three lines average diameter; for children two lines is ample. Their length should be two and one-half to three inches for an adult, and two inches for a child from six to ten years. The tent should be slightly curved and passed well back by the middle meatus of the nostril from which the blood is supposed to flow. No part of the tent should project anteriorly from the nostril, and the usual traction loop should be shortened so as to be out of way. This will lessen danger of interference by the patient. The removal should be accomplished by rotation and traction. I have noticed displacement of the Vomer or injury to the turbinated bones; a slight loss of blood is to be expected, but it soon ceases.—*Buff. Med. Jour.*

---

DILATATION OF THE URETHRA BY THE URINE ITSELF.

Towards the end of the last century Brunninghausen recommended this method of dilatation, which he claimed to be more easy and simple than that by bougies. To practice it the patient must simply compress lightly the urethra behind the glans with his fingers when ever he wishes to urinate. The pressure must be such that the urine can only escape slowly and after having remained sometime in the canal; as a necessary result, the canal will be more or less dilated through its entire length, in the constricted as well as in the healthy portion. If this be repeated every time the urine is voided, the same effects will gradually be produced as if bougies had been used, while at the same time the inconveniences of the latter were avoided. M. Berenger-Ferand has employed this method in his practice, and the following are the conclusions he has arrived at with regard to it:

1. Dilatation of the urethra by the urine, repeated at each urination for a long time after a prolonged attack of gonorrohœa, seems to prevent the formation of strictures.

2. In cases of moderate strictures it seems to have restored the normal calibre of the canal, or at least to have restored the calibre sufficiently to render micturition easy.

3. After the operation of urethrotomy it will perhaps prove useful to prevent, or at least to retard notably, the return of the constriction.

4. In case of varicose dilatations at the neck of the bladder, or in the membranous portion of the urethra, it appears calculated to be serviceable.

5. It seems to prove useful also in the cases of partial or total hypertrophy of the prostate in old men. In such patients the first drops o urine, which are emitted with so much difficulty and slowness, will serve effectually to fill the canal if the meatus be kept closed. When the ordinary calibre of the canal is once re-established in this way, the remaining contents of the bladder can be evacuated easily. The method has this great advantage, that it does away with the difficulty of emission after the first drops have escaped from the bladder; when it is not employed, the difficulty of emission persists during the entire act; the micturition, moreover, becomes intermittent, and the bladder is incompletely emptied, as a result of which, frequent desire to urinate is soon experienced.—*Lyon Medical*.

---

ELECTRIC LIGHT.

A writer thus describes the advantages of the electric light now used in Paris:

"Every sign on passing omnibus or on the buildings, every detail in the architecture of the houses, every feature of the place stands out in startling colors. The flowers are real, and the trees of lively green; every dress and hat stands out clear and sharp in its true colors as by daylight. The painted beauty of the boulevards dare not leave the gloom of the pale yellow gas. The electric light is as cruel as the sun, and her shame would be livid in the brightness. Fresh English girls, with roses and cherries won in healthful walks, stand in glad surprise under the strange white lamps, for it is sunlight, and their charms can survive the actinic test with honor. People sit in the restaurants and read their papers. It is like daylight, and it is not necessary to go to the lamp to see the print.

In a sanitary aspect it is also greatly to be preferred to gas or other lights,

"Every cúbic foot of illuminating gas burnt consumes from two to two and one-half cubic feet of oxygen, and produces two cubic feet of carbonic acid hourly. An ordinary burner consumes about forty-five cubic feet of oxygen, or as much as three full-grown men every hour. And besides the consumption of oxygen and substitution of carbonic acid in its place, owing to common impurities, illuminating gas not unfrequently gives off sulphurous acid, and sometimes the still more deadly poison, carbonic oxide. These impurities, added to the heat and moisture common to the excessive use of gas in churches, opera-houses, theatres, and other large assembly buildings and halls, and various working establishments, explain the terribly oppressive stuffiness commonly experienced in such places, and accounts for various ill effects well known to all observing physicians. Other means of lighting hitherto used, for equivalent amounts of light, are equally deleterious."

### ALL OF THE METRIC SYSTEM REQUIRED IN MEDICINE.

From the Lancet & Clinic.

To write prescriptions in Metric terms it is necessary to learn the following, only:

1 Gram is equal to 15 grains, ($\frac{15}{1}$)

1 Cubic-centimeter is equal to 15 minims, ($\frac{1}{4}$ fl. dr.)

1 Grain is equal to 0.066 ($\frac{1}{15}$) gram.

1 Fluid drachm is equal to 4 Cubic-centimeters.

Hence—

1. To convert troy grains into grams, or minims into cubic-centimeters:
   a. Divide by 10, and from the quotient subtract one-third; or, b. divide by 15; and
2. To convert apothecaries' drachm into grams, or fluid drachms into cubic-centimeters:

Multiply by 4.

The "Gram" and "Cubic-centimeter," only, should be used — (abbreviated "Gm." and "C.C.")

The centigram will be used only in books and in speaking.

All other terms, and unites, and prefixes, used in the Metric system may be wholly ignored.

The Gram and Cubic-centimeter when referring to liquids may be considered as equal quantities, except the liquids be very heavy or very light.

The average "drop" (water) may be considered equal to 0.05 C.C., or 0.05 Gm.

An average teaspoon holds 5 C.C., and an average tablespoon 20 C.C.

Example of a Metric prescription:

| | | | |
|---|---|---|---|
| R.—Hydrarg. chloridi corros...... | 0 | 25 | *Gm.* |
| Potassii iodidi.................. | 10 | 00 | *Gm.* |
| Aquæ ........................ | 100 | 00 | *C.C.* |
| Tinct. chinch. comp ........... | 100 | 00 | *C C.* |

M.

The use of a decimal line prevents possible errors.

The U. S. Marine Hospital Service has officially adopted the Metric system, and many medical societies and journals advocate its general recognition,

OSCAR OLDBERG, Phar. D.,

Washington, D. C.

### TREATMENT OF GANGLION.

Dr. Bidder (*Cbl. f. Chir.*) recommends the injection of carbolic acid. An ordinary hypodermic syringe, having a sharp needle with a cutting edge near the point, is filled with a two or three per cent. solution of carbolic acid. A fold of the skin being pinched up, the needle of the syringe is thrust under it until the point reaches the capsule of the ganglion. A little slit is made through this with the sharp-edged point of the needle, and then, the latter being slightly withdrawn, the contents of the ganglion are expressed into the surrounding tissues. The point of the needle is then once more inserted into the now emptied ganglion, and a few drops of the carbolic acid solution are injected, and a simple water dressing is afterwards applied.—*Canada Med. Record.*

### TURPENTINE AS AN EXTERNAL APPLICATION IN SMALL POX.

Dr. Farr, of Lambeth, ascribes great value to turpentine as an external application in small pox. He claims that it at once relieves any smarting or irritation, effectually corrects the unpleasant odor given off in the more confluent form of the disease, and seems in a marked degree to arrest pustulation, thereby modifying and sometimes entirely preventing pitting. In consequence of its powerful antiseptic and disinfectant properties, it tends, moreover, to prevent the spread of the infection. Mr. Farr uses it in the proportion of one part of rectified spirits of turpentine to three or four of olive oil, and applies it night and morning by means of a feather.—*The Lancet,* May 11th.

